# Prenatal Hypoxia Affects Foetal Cardiovascular Regulatory Mechanisms in a Sex- and Circadian-Dependent Manner: A Review

**DOI:** 10.3390/ijms23052885

**Published:** 2022-03-07

**Authors:** Hana Sutovska, Katarina Babarikova, Michal Zeman, Lubos Molcan

**Affiliations:** Department of Animal Physiology and Ethology, Faculty of Natural Sciences, Comenius University, 842 15 Bratislava, Slovakia; sutovska6@uniba.sk (H.S.); babarikova3@uniba.sk (K.B.); lubos.molcan@uniba.sk (L.M.)

**Keywords:** prenatal hypoxia, prenatal programming, cardiovascular system, foetus, placenta, circadian variability

## Abstract

Prenatal hypoxia during the prenatal period can interfere with the developmental trajectory and lead to developing hypertension in adulthood. Prenatal hypoxia is often associated with intrauterine growth restriction that interferes with metabolism and can lead to multilevel changes. Therefore, we analysed the effects of prenatal hypoxia predominantly not associated with intrauterine growth restriction using publications up to September 2021. We focused on: (1) The response of cardiovascular regulatory mechanisms, such as the chemoreflex, adenosine, nitric oxide, and angiotensin II on prenatal hypoxia. (2) The role of the placenta in causing and attenuating the effects of hypoxia. (3) Environmental conditions and the mother’s health contribution to the development of prenatal hypoxia. (4) The sex-dependent effects of prenatal hypoxia on cardiovascular regulatory mechanisms and the connection between hypoxia-inducible factors and circadian variability. We identified that the possible relationship between the effects of prenatal hypoxia on the cardiovascular regulatory mechanism may vary depending on circadian variability and phase of the days. In summary, even short-term prenatal hypoxia significantly affects cardiovascular regulatory mechanisms and programs hypertension in adulthood, while prenatal programming effects are not only dependent on the critical period, and sensitivity can change within circadian oscillations.

## 1. Introduction

The cardiovascular system is a dynamic system that can adapt to adverse conditions to maintain and satisfy homeostasis in the organism. Prolonged cardiovascular adaptations can result in the development of hypertension and other cardiovascular diseases in adulthood; however, hypertension can have its origins in the prenatal period when the cardiovascular system develops structurally and functionally [1]. This phenomenon observed in animals and humans is described as prenatal programming [2,3].

Prenatal programming is defined as a response to adverse factors acting during a critical prenatal period, leading to changes in the developmental trajectory with a permanent effect on the offspring’s adult phenotype [3]. Insults during the prenatal period lead to permanent structural or functional changes of the tissues and organs [3]. In general, if the prenatal factor acts during the early phase of an organ’s development, it leads to structural defects. In contrast, the action during the later phases of development affects functions [4]. The effect of prenatal insults on the foetus depends not only on the developmental stage but also on the type of insult. Consequently, some types of insults, such as various agents (warfarin, thalidomide, tetracycline, alcohol) and environmental factors of chemical (toxic metals), physical (ionising radiation) or biological origin (infection diseases), can disrupt the normal in utero development of the foetus and increase the risk of congenital disabilities, malformations, and in some cases, even death of the developing foetus [5].

Moderate effects on the foetus include high or low food availability, oxygen deficiency (hypoxia), maternal obesity, inadequate prenatal care, maternal stress, and maternal chronic diseases. The consequences of these factors can be identified during pregnancy screening and immediately after birth because they are often associated with a reduction in birth weight and asymmetric organ growth [6]. According to Barker’s theory, neonates with reduced birth weight have a higher incidence of stroke and coronary heart disease in adulthood [7]. A reduction in birth weight of neonates is the most accessible marker of poor in utero development. Indeed, many studies have recently explored an association between intrauterine growth restriction and an increased postnatal risk of cardiovascular diseases [8,9,10].

However, prenatal insults do not necessarily have to be manifested by low birth weight (Table 1) and still can lead to the programming of diseases in adulthood. This phenomenon is observed after exposure to prenatal hypoxia, which is a common complication in gravidity. In this case, neonates are born seemingly healthy, avoiding the early screening of diseases; however, due to prenatal hypoxia, the foetus has impaired endothelial function and undergoes oxidative stress [2,9,11], morphological changes in the heart and blood vessels [2,12] and changes in the activity of the autonomic nervous system [13,14], which is one of the key factors in the development of hypertension [15]. Prenatal hypoxia contributes to the development of hypertension, ischemic heart disease, coronary heart disease, heart failure, metabolic syndrome, and increased susceptibility to ischemic injury in humans [16,17].

**Table 1:** The effect of prenatal hypoxia on birth body weight.

Oxygen	Duration; Time	Animal Model	Birth Body Weight	Ref.
6.5%	1–20 ED; 8 h per day: 80 s hypoxia and 120 s normoxia; 18 cycles per hour	Sprague Dawley rats	↓	[18]
7%	13–14 ED; 3 h	Wistar rats	↓	[19]
7%	18 ED; 3 h	Wistar rats	=	[20]
9%	15–21 ED; 6 h per day	Sprague Dawley rats	=	[21]
9.5–10%	12, 24, 48, 120 h immediately prior to delivery at term	Sprague Dawley rats	↓	[22]
10%	5–19 ED	Sprague Dawley rats	↓	[23]
10%	5–20 ED	Sprague Dawley rats	↓	[13,24,25,26,27]
10%	15–20 ED	Wistar rats	↓	[28,29]
10%	from 121 ED–NA	sheep	=	[30]
10 ± 0.5%	5–20 ED	Sprague Dawley rats	↓	[31]
10.5%	15–20 ED; 4 h per day	Sprague Dawley rats	=	[32]
10.5%	4–21 ED	Sprague Dawley rats	↓	[33]
10.5%	15–21 ED	Sprague Dawley rats	↓	[34,35]
10.5%	11–17.5 ED	BALB/c mice	↓	[36]
10.5%	last 15 days of gravidity	guinea pigs	↓	[37]
10 ± 1%	7–21 ED; 3 h per day	Sprague Dawley rats	↓	[38]
11%	15–21 ED	rats	↓	[39]
11.5%	13–20 ED	Sprague Dawley rats	↓	[40]
12%	15–19 ED	Sprague Dawley rats	=	[41]
12%	14.5–21 ED	CD-1 mice	↓	[11]
13%	6–20 ED	Wistar rats	=	[2,12,42]
13–14%	6–20 ED	Wistar rats	=	[43]
14%	6–18 ED	C57BL/J6 mice	=	[44]
15%	19 ED–delivery; 10 min; 6 times per day	Sprague Dawley rats	=	[45]
NA	NA	Jackson Black C-57 mice	=	[46]
280–300 mmHg; 8000 m above sea level	14 ED–delivery; 2 h per day	C57BL/6 mice	=	[47]
PaO_2_ 13 mmHg	14 days	sheep	=	[48]
3820 m above sea level	30–120 ED	sheep	=	[49]
4000 m above sea level on first day, 5000 m above sea level on the second to fifth day	14–18 ED, 8 h per day	rats	↓	[50]
chronic anaemia	NA	sheep	=	[51]
9000 m above sea level; PaO_2_ 42 mmHg	14–19 ED, once 4 h	albino rats	↓	[52]
NA	105–138 ED	sheep	↓	[53]

ED, embryonic day; NA, non-available; ↓, decreased birth body weight; =, no changes of the birth body weight in comparison with control group.

The most important signalling molecules that respond to changes in oxygen content are hypoxia-inducible factors (HIFs) from the family of heterodimeric transcription factors. The HIF transcription factor is composed of two subunits, α and β. Under normoxia, HIF-1α is rapidly degraded, but if the partial pressure of oxygen decreases, it is stabilized and accumulates in the cells. HIF-1α maintains oxygen homeostasis by regulating the expression of hundreds of genes and interacts with pathways involved in the regulation of the cardiovascular system, such as the chemoreflex, sympathetic drive [54], renin-angiotensin-aldosterone system (RAAS) [55,56], and local vessel wall components such as nitric oxide (NO) [57,58,59] and endothelin-1 [60,61]. Moreover, HIF directly interacts with the circadian clock because HIF-1α controls the expression of several canonical circadian genes and the response to hypoxia is gated by the circadian clock [62]. The relationship between the HIF and clock can be reciprocal, because in several experimental models, hypoxia was found to reduce the amplitude of circadian rhythms or their response to phase shifts [63].

In this review, we analyse the effects of prenatal hypoxia, which is not predominantly associated with intrauterine growth restriction but leads to changes in blood pressure and hypertension in adulthood. The effects of intrauterine growth restriction interfere with metabolism and can lead to multifactorial changes in the development of cardiovascular regulatory mechanisms and programming blood pressure. In our review, we aim to describe: (1) The response of cardiovascular regulatory mechanisms, such as the chemoreflex, adenosine, NO, reactive oxygen species (ROS) and RAAS on prenatal hypoxia and its effects on hypertension in adulthood. In addition, we describe the effects of prenatal hypoxia on morphological-functional changes in the heart, vessels, and kidneys, which can be risk factors for hypertension. (2) The role of the placenta in causing and attenuating the effects of hypoxia. (3) The availability of oxygen to the foetus can be affected by the mother’s current health status and environmental conditions, thus, the mother plays a key role in causing prenatal hypoxia. (4) The sex-dependent effects of prenatal hypoxia on cardiovascular regulatory mechanisms while also considering the circadian sensitivity of foetuses to prenatal hypoxia within 24 h.

## 2. Methodology

This review includes publications describing the effects of prenatal hypoxia on foetal cardiovascular regulatory mechanisms, which are not associated with intrauterine growth restriction in a sex- and circadian-dependent manner. The literature research was performed in the PubMed, Scopus and Google Scholar databases, using the following keywords: “prenatal hypoxia”, “antenatal hypoxia”, “cardiovascular system”, “blood pressure”, “foetus”, “prenatal programming”, “chemoreflex”, “adenosine”, “nitric oxide”, “reactive oxygen species”, “catecholamines”, “angiotensin”, “heart”, “blood vessel”, “kidney”, “sex”, “male”, “female”, “placenta” and “circadian variability”. Relevant studies were evaluated by title and abstract, followed by a full-text overview. We analysed the effects of prenatal hypoxia on cardiovascular regulatory mechanisms predominantly in mice, rats, and sheep. We present publications (Table 1) that describe the prenatal hypoxia effects on body weight often independent of intrauterine growth restriction and we included in our review all papers that were published by September 2021.

## 3. Prenatal Hypoxia

**Figure 1:**
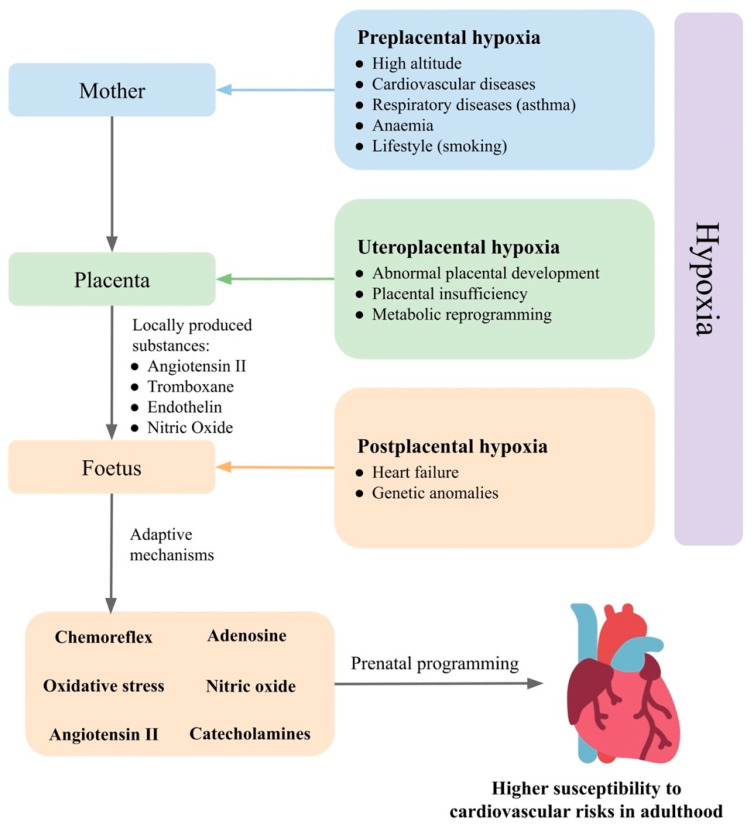
Prenatal hypoxia as result of mother, placenta, and foetus and their effects on cardiovascular regulatory mechanisms in the foetus. Based on the level in which prenatal hypoxia occurs, it can be divided into preplacental hypoxia, uteroplacental hypoxia, and postplacental hypoxia. The foetus is able to compensate for oxygen deficiency and maintain homeostasis by the activation of regulatory mechanisms. The response of the foetus to prenatal hypoxia can also be modulated by the placenta and the mother. Changes in the set points of the cardiovascular regulatory mechanisms in the foetus increase susceptibility to hypertension in adulthood.

Prenatal hypoxia is a common complication in pregnancy, developing from various causes (Figure 1). In gravidity, prenatal hypoxia often occurs as a comorbidity of maternal diseases, such as hypertension, anaemia and respiratory diseases or as a consequence of poor maternal lifestyle, such as smoking, so-called preplacental hypoxia. Prenatal hypoxia can also develop as a consequence of morphological or functional changes in the placenta, such as failed remodelling of spiral arteries or metabolic reprogramming (uteroplacental hypoxia). Disturbed fetoplacental perfusion due to changes in foetal circulation or genetic anomalies in the circulatory system can lead to postplacental hypoxia [64].

### 3.1. Foetus

Foetal blood is hypoxic compared with maternal blood. The foetal partial pressure of oxygen is reduced to 20–30 mmHg, while in the mother, the arterial partial pressure of oxygen is about 75–100 mmHg at sea level [65,66,67]. Nevertheless, the foetus can cope with up to a 50% (10–15 mmHg) reduction of the partial pressure of oxygen [68]. This has been well-described in sheep used as a common animal model of prenatal hypoxia. In sheep, during normoxia, the maternal partial pressure of oxygen is about 100 mmHg. In comparison, during hypoxia, it can decrease up to 35 mmHg, and the foetus can compensate for this condition. In the case of a sheep foetus, due to hypoxia, the oxygen saturation can decrease from 20 mmHg to 10.5 mmHg in the umbilical artery and from 34 mmHg to 15 mmHg in the umbilical vein [69]. A decrease of the partial pressure of oxygen in the foetus causes femoral vasoconstriction because of local and neurohumoral regulatory mechanisms [70], which can cooperate with the foetal, placental, or maternal regulations. 

The adaptive response to hypoxia is a combination of the following five factors:

(1) Reflex response mediated by the chemoreflex and α_1_-adrenergic signalling in peripheral vessels [70,71,72]. Hypoxia also directly affects chromaffin cells in the adrenal medulla, thereby stimulating the release of catecholamines. Chromaffin cells of the adrenal medulla have a chemosensitive function until a sympathetic innervation develops between the adrenal glands and the central nervous system [73].
(2) An endocrine response that is involved in maintaining peripheral vasoconstriction and is activated within about 15 min of the onset of hypoxia, including angiotensin II (Ang II) and other vasoactive substances [71,74,75].
(3) Local components that respond to the direct effect of hypoxia at the tissue level. The vascular endothelium acts as a hypoxic sensor but also an effector system that releases NO [76], adenosine [77], and endothelin-1 [78], thus affecting the function of vascular smooth muscle.
(4) Placental vasoactive substances are released from the placenta into the foetus and can thus modulate the response to hypoxia. Such substances include adenosine [79], Ang II, thromboxane, endothelin, NO [80], glucocorticoids and others [81].
(5) Maternal factors that pass from the mother to the foetus. Environmental hypoxia, during which both the mother and the foetus are hypoxic, causes adaptive changes not only at the level of the foetus but also in the mother. Some of the mother’s adaptation mechanisms may also affect foetal development [81].

Activation of the described mechanisms as a response to prenatal hypoxia ensures sufficient blood flow to vital organs, such as the brain and heart, by peripheral vasoconstriction and reduction in oxygen consumption by the tissues and organs. We further describe the effects of prenatal hypoxia on cardiovascular regulatory mechanisms: the chemoreflex, adenosine, NO, ROS, and RAAS, as well as the effects of prenatal hypoxia on morphological-functional changes in the heart, blood vessels, and kidneys (Table 2).

**Table 2:** Effects of prenatal hypoxia on the regulatory mechanism of blood pressure.

	Prenatal Hypoxia Type	Animal Model	Hypoxia Outcomes	Ref.
Adenosine	Arterial PaO_2_ 15 mmHg; 1 h	Sheep	Foetal acidosis, mean arterial pressure increase, a transient heart rate decrease	[77]
	Hypoxia/anoxia; 20 or 60 min	A_1_R^+/+^, A_1_R^+/−^ and A_1_R^−/−^ C57BL mice, hippocampal slices, isolated brainstem spinal cord	Reduction in field excitatory postsynaptic potential	[82]
	10% O_2_; 7.5–10.5 ED	A_1A_R^+/+^ and A_1A_R-deficient C57BL/6 mice	Growth retardation, less stabilized HIF-1α protein and cardiac gene expression in *A_1A_R^−/−^* embryos	[83]
	10–12% O_2_; 30 min; 122–128 ED	Western sheep	Cortical blood flow increase, attenuated by a non-selective adenosine receptor antagonist	[84]
NO, ROS	13% O_2_; 6–20 ED	Wistar rats	Foetus: aortic thickening, enhanced nitrotyrosine staining and increased cardiac HSP70 expression. Adult offspring: impaired NO-dependent relaxation, increased myocardial contractility	[2]
	12% O_2_; for 4, 7, or 10 days; 58–62 ED	Hartley-Duncan guinea pigs	Increased eNOS mRNA in foetal ventricles, not altered K^+^-channel activation in response to acetylcholine-stimulated coronary dilation	[59]
	40–50% uteroplacental artery ligation; 25 ED	New Zealand white rabbits	Normal left and right ventricular thickness, increased vessel dilatation; HIF-1α, eNOS, p-eNOS, and iNOS induction suggesting increased NO and oxidative stress in the hearts	[85]
	13% O_2;_ most of gestation (prior to day 5)	Wistar rats	Maternal and placental oxidative stress—prevented by maternal treatment with vitamin C	[42]
	13% O_2_; 6–20 ED	Wistar rats	Increased LF/HF HRV ratio and baroreflex gain—prevented by vitamin C	[86]
	Acute: 10% O_2_; 0.5 h, 127 ± 1 ED; chronic: 10% O_2_; 105–138 ED	Welsh Mountain sheep	Mitochondria-derived oxidative stress, endothelial dysfunction and hypertension in adult offspring	[53]
	6% O_2_; 0.5 h	Welsh Mountain sheep	Increased redistribution of blood flow and the glycemic and plasma catecholamine responses	[87]
	14 ± 0.5% O_2_; 1–19 ED (embryos underwent euthanasia)	Bovans Brown eggs	Cardiac systolic dysfunction, impaired cardiac contractility and relaxability, increased cardiac sympathetic dominance, endothelial dysfunction in peripheral circulations	[88]
	Conceived, gestated, born and studied at Putre Research Station (3600 m above sea level)	Sheep (neonates)	Worsened carotid blood flow, vascular responses to potassium, serotonin, methacholine, and melatonin; diminished endothelial response via NO-independent mechanisms in isolated arteries	[89]
	10.5% O_2_; 15–21 ED	Sprague Dawley rats	Revealed reprogramming of the mitochondrion	[90]
	11% O_2_; 15–21 ED	Sprague Dawley rats	Male and female foetuses: increased oxidative stress in placentas; 7-month-old male and female offspring: cardiac diastolic dysfunction; 13-month-old female offspring: reduced vascular sensitivity to methacholine, 13-month-old male offspring: decreased vascular sensitivity to phenylephrine	[91]
	13–14% O_2_; 6–20 ED	Wistar rats	Increased α1-adrenergic reactivity of the cardiovascular system, enhanced reactive hyperemia, sympathetic dominance, hypercontractility and diastolic dysfunction in the heart	[92]
	7% O_2_; 2 h; 50–55 ED; foetal hearts were harvested at the end of hypoxia	Guinea pigs	Decreased heart ATP, lipid peroxides, 4-hydroxynonenal and malondialdehyde; increased apoptotic index, unremarkable foetal heart morphology, normal postpartum neonatal cardiac function and cerebral histology	[93]
	Acute: 220–240 mmHg; 10,000 m above sea level; 4–5 min; 18 ED–delivery; chronic: 280–300 mmHg; 8000 m above sea level); 2 h; 14 ED–delivery	C57BL/6 mice	Acute hypoxia: decreased basal O_2_ consumption rate and intensity of oxidative phosphorylation by the brain mitochondria of newborn, the activation of the respiratory complex II; chronic hypoxia: increased basal O_2_ consumption rate and oxidative phosphorylation intensity	[47]
RAAS	10.5% O_2_; 6–21 ED	Sprague Dawley rats	Foetal growth restriction, impaired trophoblast invasion and uteroplacental vascular remodeling, increased plasma ET-1 levels, *prepro-ET-1* mRNA, ET-1 type A receptor and AT_1_ receptor in the kidney and placenta	[78]
	12% O_2_; from 14.5 ED	CD1 mice	Weaning: both sexes: increased susceptibility to salt-induced cardiac fibrosis; male: renal fibrosis by high salt, increased renal *renin* mRNA; 12 months: both sexes: increased renal *renin* mRNA expression and concentrations, male: increased *AT_1a_* mRNA expression	[94]
	10.5% O_2_; 4–21 ED	Sprague Dawley rats	Increased superoxide production and decreased SOD expression, enhanced NADPH4, but not NADPH1 or NADPH2 in foetal aortas; increased Ang II-mediated vessel contractions in foetal thoracic aortas blocked by losartan	[33]
	Acute isocapnic hypoxaemia (foetal PaO_2_ 12–14 mmHg); 1 h; 110/114–124/128 ED	Sheep foetuses	No effects in foetal heart rate, mean arterial pressure, baro- or chemoreflexes, femoral blood flow, femoral vascular resistance or foetal growth	[48]
Reflex	Aortic PaO_2_ 12–15 mmHg without alterations in foetal PaCO_2_; 1 h; 124 ED	Welsh Mountain sheep foetuses	Transient bradycardia, femoral vasoconstriction and increases in plasma noradrenaline and adrenaline; the NO clamp: persisted bradycardia, greater peripheral vasoconstrictor and catecholaminergic responses—enhanced the chemoreflex sensitivity	[70]
	PaO_2_ 15 mmHg; 137–144 ED	Border Leicester Merino cross sheep	Reduced and delayed the *I_A_*-type current	[73]
	Aortic PaO_2_ 10–11 mmHg without alterations in foetal PaCO_2_; 1 h; 117–118 ED	Sheep foetuses	Bradycardia, increased arterial blood pressure, femoral vasoconstriction, blood glucose, lactate concentrations, plasma epinephrine and norepinephrine	[95]
	Foetal arterial oxygen saturation by 47.3% (uterine blood flow restriction); 118–126 ED	Sheep foetuses	Bradycardia, not in denervated foetuses, followed by a tachycardia; increased foetal heart rate in denervated foetuses; transiently increased foetal blood pressure in intact foetuses and decrease in denervated foetuses; increased cerebral blood flow in both intact and denervated foetuses; decreased carotid vascular resistance in denervated foetuses	[96]
	10% O_2_; 5–20 ED	Sprague Dawley rats	Decreased dopamine content in the carotid bodies; until 3 weeks after birth: hyperventilation and disturbed metabolism	[31]
	10% O_2_; 5–20 ED	Sprague Dawley rats	Evaluated resting ventilation and ventilatory response; periphery: reduced tyrosine hydroxylase activity within the first postnatal week and enhanced later; central areas: upregulated tyrosine hydroxylase activity within the first postnatal week and downregulated later	[27]

ED, embryonic day; O_2_, oxygen; PaO_2_, partial pressure of O_2_; PaCO_2_, partial pressure of carbon dioxide; A_1_R, adenosine 1 receptor; HSP70, heat shock protein 70; ROS, reactive oxygen species; RAAS, renin-angiotensin-aldosterone system; NO, nitric oxide; HIF, hypoxia-inducible factor; eNOS, endothelial NO synthase; p-eNOS, phospho-eNOS; iNOS, inducible NO synthase; LF/HF, the ratio of low frequency to high frequency; HRV, heart rate variability; ET-1, endothelin-1; AT_1_, angiotensin II type 1 receptor; SOD, superoxide dismutase; NADPH, nicotinamide adenine dinucleotide phosphate oxidase; Ang II, angiotensin II.

Effects of prenatal hypoxia have been studied in different animal models, including chicken (hatching 21 days), mice (full-term 21 days), rats (full-term 22 days), guinea pigs (full-term 65 days) and sheep (full-term 147 days) [71,97] and each one has its advantages. Sheep have a relatively similar heart size compared with humans, allowing better observation of the changes in foetal cardiovascular parameters in utero. In contrast, mice and rats have smaller hearts than humans; however, they have a rapid reproduction rate, allowing a relatively fast observation of changes in the postnatal period. In addition, the use of mice in a prenatal hypoxia study allows the creation of knockouts to study selected regulatory mechanisms. The chicken embryo develops without direct humoral contacts with the mother, and the development can be easily manipulated [98]. Mice and rats are altricial species, and their development (e.g., nephrogenesis) continues after birth [99], whereas in precocial humans, sheep and guinea pigs, the development is terminated during the prenatal period [100]. The difference among animal models is also related to the time of organogenesis and the critical period of development, which can partially explain some interspecies variation in the foetal responses to prenatal hypoxia.

#### 3.1.1. Reflex Response

An important regulatory mechanism responding to changes in the partial pressure of respiratory gases (oxygen and carbon dioxide) are peripheral chemoreceptors (Table 2). Acute prenatal hypoxia activates the carotid bodies and triggers a chemoreflex response [70]. Peripheral chemoreceptors activate afferent pathways to the brainstem, where they affect the cardiovascular centres. The efferent signal from these centres is transmitted through cholinergic stimulation of the heart and through α-adrenergic stimulation of blood vessels. The result is a decrease in heart rate and an increase in peripheral vasoconstriction in acute response; however, after prolonged prenatal hypoxia, tachycardia, and blood pressure decrease [70,71,95,96]. The heart rate response to prenatal hypoxia is abolished after bilateral carotid denervation, whereas the treatment does not affect the blood pressure response [96].

Peripheral vasoconstriction due to α-adrenergic stimulation increases the right ventricular afterload and increases the blood flow through the foramen ovale. Then, blood from the right atrium enters the left atrium and the left ventricle, which increases blood flow to the ascending aorta and cerebral and coronary circulation [68]. These effects on the sympathetic nervous system and chemoreflex are mediated by the HIF-1α/HIF-2α ratio. Isolated carotid bodies from *Hif-1α*^+/−^ knockout mice did not respond to short-term hypoxia, while long-term hypoxia (repeated acute hypoxia for three days) diminished ventilatory response and impaired the sensitivity to hypoxia in Hif-1α^+/−^ knockout mice [101]. On the other hand, in the carotid bodies of *Hif-2α*^+/−^ mice, an elevated response (breathing abnormalities and elevated plasma noradrenaline levels) to acute hypoxia was observed. It seems that HIF-2 has an antagonistic role to HIF-1 in oxygen sensing by carotid bodies [102]. Complete deficiency of HIF-1α or HIF-2α is often lethal with multiple organ (including heart and vessels) malformations [103,104].

Hypoxia also directly affects chromaffin cells in the adrenal medulla, the primary source of catecholamines in the foetus. The higher heart rate after prolonged hypoxia can result from the increased catecholamine secretion [49,73]. In the foetus, the adrenal glands are the primary source of catecholamines. The adrenal medulla in the foetus is not innervated by the cholinergic splanchnic nerve; however, non-neuronal regulation is directly sensitive to hypoxia [73]. The chemosensory function of chromaffin cells in sheep includes the inhibition of potassium channels, membrane depolarization, increases in intracellular calcium concentration [73], leading to the release of catecholamines into plasma with an increased level persisting to postnatal life, while enzyme expression is also affected [25,26].

In prenatal life, L-, N-, and P/Q-type Ca^2+^ channels have a similar ratio in the influx of Ca^2+^ [73]. The function of K^+^ is age-dependent; during the initial stages of in utero development, chromaffin cells express more ATP-sensitive K^+^ currents, while in the late stages, more Ca^2+^ activated K^+^ currents are expressed [105]. Chromaffin cells lose their “chemosensitivity” ability after splanchnic innervation of the adrenal medulla. Loss of chemosensitivity is also associated with altered expression of the T- type Ca^2+^ channels [106].

Regulation of the respiratory response through the nervous system appears to be less important, as the foetus does not have its respiratory system fully developed until birth. Therefore, the response mediated by central chemoreceptors is different in foetuses and adults. While the foetus responds to acute hypoxia by inhibition of breathing, in adults, acute hypoxia causes initial hyperventilation and subsequent respiratory depression [27,31]. Prenatal hypoxia can change the maturation of these chemoreceptors, which can be manifested later in adulthood. In rats exposed to prenatal chronic hypoxia (10% O_2_) from embryonic day (ED) 5 to ED 20, a change in respiratory response to acute postnatal hypoxia (10% O_2_; 10 min) was observed as the absence of the increased respiratory rate that is typical for the first phase of the response to hypoxia [27,31].

#### 3.1.2. Adenosine

Reduced oxygen availability decreases oxidative phosphorylation in the mitochondria and stimulates the conversion of adenosine monophosphate to adenosine, the biological effect of which is mediated via four classes of receptors: A_1_, A_2A_, A_2B_, and A_3_. Activation of these receptors depends on adenosine concentration. A slight increase in the adenosine level activates the A_1_ receptor, which has the highest sensitivity. In contrast, activation of the A_3_ receptor occurs when adenosine concentration is exceptionally high in severe pathologic conditions [107]. Adenosine indicates oxygenation in the tissues and in the case of hypoxia, the adenosine level increases. Adenosine has a suppressive effect on plasma cortisol levels and the function of the adrenal cortex in foetal sheep. Therefore, adenosine can play an important role in protecting the foetus against intrauterine stress [108,109]. Adenosine also has a vasodilating effect on the coronary circulation in the sheep foetus, increasing blood flow and ensuring oxygen supply [110]. In the foetus, adenosine regulation of the heart rate is dominant compared with adrenergic and cholinergic stimulation [107]. In the foetus, exogenously elevated adenosine is followed by a decrease in heart rate to asystole, while no response is observed after the administration of drugs, which increases endogenous catecholamines or acetylcholine release [107]. The same effects are observed in hypoxia, where an increased adenosine concentration activates the A_1_ receptor and lowers the heart rate [107].

Adenosine A_1_ receptors are already expressed during the embryonic and organogenesis periods in the heart and brain [107], but they are not essential for normal foetal development in normal pregnancy. Mice deficient for A_1_ receptors showed no developmental defects, growth restriction, or changes in blood pressure and heart rate [82]; however, adenosine A_1_ receptors are important in protecting the foetus from hypoxia. Mice lacking the A_1_ receptor have more serious consequences after exposure to prenatal hypoxia, more pronounced growth restriction, and more significant morphological changes in the heart (disproportionate reduction in heart size, thinner ventricular walls) compared with A_1_^+/+^ receptor and A_1_^+/−^ receptor mice. In A_1_^−/−^ receptor mice, decreased stabilisation in HIF-1α was also observed. The reduced stabilisation in HIF-1α in A_1_^−/−^ receptor mice, resulted in the expression of genes that protect against hypoxia (adrenomedullin, carbonic anhydrase 1, and catalase) also being reduced. In contrast, in A_1_^+/+^ receptor and A_1_^+/−^ receptor mice, the expression of HIF-induced genes was up-regulated [83,111]. This points to an important link between the adenosine and HIF signalling pathways in the hypoxia in the prenatal period (Table 2).

The importance of adenosine and its receptors in prenatal programming was reported in a study with non-selective adenosine antagonists. In sheep treated with an adenosine receptor antagonist, a bradycardia response, increased blood pressure, and peripheral vasoconstriction were not observed after acute hypoxia [77,95]. Thus, adenosine receptors appear to play a significant role in mediating the chemoreflex function [95]. Denervation of peripheral chemoreceptors together with increased levels in adenosine did not reduce the heart rate during hypoxia. In this line, blockade of the adenosine receptor and functional chemoreflex also did not decrease the heart rate [77]. Except for the peripheral chemoreflex sensitivity, adenosine mediates multilevel responses. For example, adenosine receptors (A_2A_) in the brain are responsible for inhibiting breathing [84], whereas adenosine receptors in the chromaffin cells of the adrenal medulla increase catecholamine levels due to acute hypoxia.

#### 3.1.3. Nitric Oxide, A Reactive Oxygen Species 

Acute foetal hypoxia causes the dilation of blood vessels providing blood flow to vital organs, such as the heart and brain [59]. Vascular dilatation is caused by increased NO, adenosine, and prostanoids. In peripheral vessels, increased NO synthesis by endothelial NO synthase (eNOS) compensates for the vasoconstrictor responses of hypoxia (Table 2) through the actions of the chemoreflex and catecholamines [70]. The release of catecholamines is reduced by NO donors, and the inhibition of NO synthesis conversely increases the release of catecholamines from the adrenal medulla [70].

The consequences of hypoxia on the expression of eNOS are contradictory. Some studies show increased eNOS in the carotid artery and decreased in the femoral artery [57] at the protein and gene expression levels [59]. In the heart, eNOS expression was increased at the protein and decreased at the gene expression level [85]. The effect of hypoxia on eNOS expression depends on the duration of hypoxia because increased ROS production may interfere with the bioavailability of NO during prolonged hypoxia [58]. The dynamic relationship between ROS and NO determines vascular tone because the hypoxia-induced increase in ROS, and thus the foetal ROS/NO ratio, potentiates peripheral vasoconstriction and redistribution of blood flow from the peripheral circulation to the vital organs, such as the brain and heart [112]. Nicotinamide adenine dinucleotide phosphate (NADPH) oxidase-derived ROS can react with NO to form a stable peroxynitrite anion, reducing the bioavailability of NO. Exposure to prenatal hypoxia increases the expression of NADPH homologue 1, which stimulates the production of superoxide and impairs endothelium-dependent vasodilatation [113]. The importance of oxidative stress in prenatally programmed hypertension is demonstrated by studies in which antioxidants, such as vitamin C and melatonin, have been administered [2,42,86]. Vitamin C decreased nitrotyrosine and HSP 70 in the aorta [2], increased the bioavailability of NO, as well as decreased HSP 70 and increased HSP 90 in the placenta [42]. Vitamin C also reduced sympathetic to parasympathetic power, thereby weakening peripheral vasoconstriction, and affecting the brain-sparing effect [53,86]. Melatonin is a pleiotropic compound, because in addition to its role in circadian regulation, it has strong antioxidant effects. Melatonin reduces the mean arterial blood pressure [87], improves baroreflex control [114], and, in general, can improve cardiac function as myocardial relaxation, myocardial contractility, and left developed ventricular pressure [88]. As a result of its ability to reduce oxidative stress, melatonin improved endothelial function, thereby exhibiting its vasodilatory effects [88,89]. In addition, melatonin also modulates activity of the autonomic nervous system, reduces adrenergic activation, and increases cholinergic stimulation, thereby reducing blood pressure [87,114]. The protective effects of melatonin, vitamin C, and other antioxidants support the hypothesis that oxidative stress plays an important role in mediating the effects of prenatal hypoxia on the foetus, and those antioxidants can reduce the stress consequences on the cardiovascular system in adulthood.

Decreased oxygen concentration leads within a few minutes to the suppression of anabolism, stimulation of anaerobic glycolysis, and inhibition of aerobic metabolism in mitochondria [109]. The importance of phenotypic reprogramming of mitochondria in the heart after an insult is known in adults and can also be important for the prenatal programming of heart disease in adulthood (Table 2) [90]. The significance of mitochondria function in cardiovascular programming is demonstrated by using MitoQ in the treatment of prenatal hypoxia [53,91,92]. Administration of MitoQ, which acts downstream from superoxide production, prevents the harmful lipid peroxidation and mitochondrial damage initiated by superoxide. Studies point to the importance of mitochondria in cardiovascular disease programming, maintaining the brain-sparing effect and decreasing the risk of cardiovascular disease. Changes in mitochondrial activity depend on the duration of prenatal hypoxia. Acute prenatal hypoxia, induced at ED 18 for 2 h in mice, decreased the basal oxygen consumption and oxidative phosphorylation in the mitochondria of neonates. After acute prenatal hypoxia in guinea pigs, cardiac level of ATP at the end of hypoxia were significantly reduced, but no changes in cardiac metabolism were observed in neonates [93]. Chronic prenatal hypoxia, from ED 14 until delivery, increases the basal oxygen consumption and oxidative phosphorylation in the central nervous system of neonates. Chronic prenatal hypoxia increases the expression of *Nfe2l2* (Nuclear factor erythroid-derived 2-like 2), which is responsible for protection against oxidative stress and mitochondrial oxidative stress, with no effect on antioxidant enzyme expression in the foetal heart. In contrast, in the adult offspring rats, an increased expression of *Nfe2l2*, catalase and glutathione peroxidase was observed in the heart as a compensatory antioxidant response to prenatal hypoxia [92]. These results indicate that chronic prenatal hypoxia leads to the adaptation of the mitochondrial function in a duration-dependent manner [47].

#### 3.1.4. Renin-Angiotensin-Aldosterone System

The renin-angiotensin-aldosterone system is important in regulating blood pressure, electrolytes, and water homeostasis. Decreased renal blood flow induces the release of renin from juxtaglomerular cells and the conversion of inactive angiotensinogen to angiotensin I, which is converted to Ang II by the angiotensin-converting enzyme. The biological effects of Ang II are mediated through AT_1_ and AT_2_ receptors and the RAAS functions differ between adults and the developing foetus [115]. In the adults, the RAAS is important for long-term regulation of blood pressure and its effects are mediated by AT_1_ receptors via peripheral vasoconstriction, fluid balance, and electrolyte control, whereas the AT_2_ receptor is responsible for a counterregulatory role through stimulation vasodilatation, natriuresis and inhibition proliferation and cell differentiation [116]. During the prenatal period, both AT_1_ and AT_2_ receptors are present and are involved in the control of neural differentiation, cell apoptosis and cell proliferation [115]. Exposure to prenatal hypoxia results in cardiac fibrosis and hypertrophy due to an adaptive response [117] and activation of the signalling pathways related to the AT_1_ receptor and the transforming growth factor-β1. Angiotensin II stimulates the synthesis of collagen and extracellular matrix proteins through activation of the AT_1_ receptor and transforming the growth factor-β1 [117]. The development of fibrosis and cardiac hypertrophy in foetuses exposed to prenatal hypoxia can be amplified by increased salt intake, which only supports the role of RAAS in the development of these morphological changes [94]. Moreover, AT_1_ and AT_2_ receptors play a significant role in the prenatal programming of blood pressure and setting the set points of its regulatory mechanisms (Table 2) [33,94]. This conclusion is supported by using RAAS blockers, which reduce blood pressure and eliminate the effects caused by prenatal hypoxia in the cardiovascular system [118]. Angiotensin II receptors are important for the development of nephrons and the kidneys, can increase sympathetic nervous activity, oxidative stress, and cause cardiac and renal fibrosis associated with the development of hypertension and other cardiovascular diseases.

Hypoxia-induced HIF-1α stimulates the expression of AT_1_ receptors and the angiotensin-converting enzyme [55], while silencing of HIF-1α suppressed Ang II-mediated effects in the kidney [56]. In foetal thoracic aortas of rats (ED 21), losartan, the AT_1_ receptor antagonist, suppressed Ang II-mediated vessel contractions induced by hypoxia via the NADPH oxidase 4-dependent pathways [33], whereas the effect of hypoxia was more pronounced in rats when offspring were exposed prenatally to a high salt diet [94], suggesting the participation of structural changes in the kidney. The experiments indicate a strong interaction of RAAS and prenatal hypoxia in the development of hypertension [78].

Acute hypoxia in the middle of pregnancy causes short-term changes in the kidneys of sheep. These short-term compensatory mechanisms, such as glomerular hypertrophy, allow renal function to be maintained, but nephron deficiency later in life may lead to an inability to maintain fluid homeostasis and increase the susceptibility to hypertension and renal disease. Decreased nephron formation may be due to impaired morphogenesis, increased apoptosis, and changes in RAAS activity. The critical period of nephron formation depends on the animal model and is found in the interval from the first trimester to the early postnatal stage [119]. Decreased nephron formation may also be due to growth factors, because in vitro studies showed that transforming growth factor-β1 reduced the nephron number [120], inhibited ureteric branching and affected tubular development [121]. Increased expression in transforming growth factor-β1 was also observed in other “a poor in utero environment” models and was associated with a reduced number of nephrons [119,122]. Repeated exposure to intermittent hypoxia for two weeks in sheep did not lead to a change in body or organ weight, except for the kidneys, which were smaller in sheep exposed to hypoxia, and the reduction in kidney weight may be due to decreased renal blood flow and growth factors [48]. A decreased nephron number and reduced weight of the kidneys are often associated with intrauterine growth restriction; however, fewer nephrons have also been observed in children with a “normal” birth weight. This may be of clinical significance because a decreased nephron number and “normal” birth weight are associated with sudden infant death syndrome [48,123].

#### 3.1.5. Morphological-Functional Changes in the Heart and Blood Vessels

Prenatal hypoxia causes peripheral vasoconstriction and bradycardia. As a result, pressure and volume overload of the heart occurs and leads to heart remodelling. The impairment of prenatal development can induce changes in the development of the heart conduction system, which is associated with sudden infant death syndrome. Disorders at the level of the conduction system can reflect deregulated atrial, atrioventricular node, or conduction pathways and mutations in sodium channels [124,125]. If prenatal hypoxia is prolonged, the heart becomes hypertrophied to maintain its function [126]. As a result of short-term hypoxia in the last trimester of pregnancy, an increase in mononuclear cardiomyocytes was observed in both the right and left ventricles and was accompanied by increased collagen deposition [52]. Moreover, the elevated length to width ratio of cardiomyocytes was found in the right ventricle [52] and similar results in heart development were observed after chronic prenatal hypoxia [127]. Development of the cardiomyocytes is regulated by Ang II, cortisol, and insulin-like growth factor I and II [110,128]. Prenatal hypoxia can affect the development of cardiomyocytes through these factors and has an important role in oxidative stress [52]. The observed changes can cause cardiac dysfunction under pathological conditions and ischaemia-reperfusion injury [34].

Prenatal hypoxia between ED 11.5–13.5 causes the most significant changes in the heart, compared with the brain and spinal cord of mice foetuses. As a result of the decreased cardiomyocyte proliferation, the ventricular myocardium was thin, the epicardium was detached, and the ventricular mass and wall thickness was reduced. For example, sheep foetuses affected by prenatal hypoxia had smaller myocytes than the controls [129]. In general, prenatal hypoxia slows heart maturation [130] and these morphological changes accompany reduced cardiac output and diastolic function [129].

Exposure to chronic hypoxia (10 ± 1% O_2_) from ED 7 to ED 21 in rats increased the relative weight of the heart and brain [38]. Another study with chronic hypoxia (13% O_2_) from ED 6 to ED 20 in rats did not show a change in heart weight or morphological changes in the ventricles; however, isolated offspring hearts had increased ventricular contractility (dP/dt_max_), as well as a suppressed chronotropic response to a muscarinic agonist and an increased response to a β_1_-adrenoreceptor agonist due to chronic prenatal hypoxia [2]. Meanwhile, another study in sheep showed that prenatal hypoxia from ED 103 (full-term 147 days) increased the left ventricular end-diastolic pressure and decreased myocardial contractility and relaxation in the isolated heart [131]. Moreover, a study associated with intrauterine growth restriction induced by intermittent hypoxia from ED 14 to ED 18 showed no changes in dP/dt_max_ on ED 22 and postnatally; however, these rats had an increased inotropic response [50]. On the other hand, in rats exposed to 12 h of hypoxia on ED 20, no differences in heart rate and blood pressure response to noradrenaline compared with the control rats were observed [132], thus, it seems that changes are prenatal hypoxia duration dependent.

Prenatal hypoxia also increases the aortic wall thickness [2,28]. In 16-month-old rats, prenatal hypoxia impaired the endothelial function, thickening and deposition of fibrils in the intima, and the migration and proliferation of vascular smooth muscle cells to the intima [38]. Morphological changes of the heart and blood vessels can be associated with an increased load on the heart and blood vessels and are likely to be mediated by oxidative stress and endothelial dysfunction [2,75,131]. Treatment with vitamin C [2], the NADPH inhibitor apocynin, and superoxide dismutase [9] eliminated the adverse effects of prenatal hypoxia on endothelial dysfunction and morphological changes of the cardiovascular system; however, administration of MitoQ improved the endothelial function in rats exposed to prenatal hypoxia but did not normalise hyperactivity of the sympathetic nervous system [14,133]. These findings point to the complexity of the mechanisms involved in the prenatal programming of the cardiovascular system.

### 3.2. Placenta

Maternal and foetal blood do not mix, but their circulations are in close proximity in a newly formed fetomaternal interface, the placenta. This transient organ provides the environment for the exchange of nutrients and gases between the mother and the foetus and protects the foetus from deleterious environmental factors [134]. Oxygen crosses the placental barrier by simple diffusion down its concentration gradient, so the efficiency of its transport depends mostly on uterine blood flow, placental morphology, and placental metabolism [135]. During pregnancy, uteroplacental blood flow increases several times to meet foetal demands and, therefore, structural changes must constantly occur in the placenta [136,137]. If the placenta is exposed to adverse effects, such as hypoxia, its structure and function must change, thus sparing the developing foetus from oxygen deprivation [138]. On the other hand, when placental development is disturbed, the placental oxygen supply might become limited [43,139]. 

Placental function is linked to its structure. In humans, maternal spiral arteries deliver oxygenated blood into the space between placental chorionic villi, so the villous brush border membrane is washed directly by maternal blood. On the foetal side of the placenta, chorionic villi encompass foetal capillary networks. Maternal blood is thus separated from foetal circulation by several tissue layers [140]. Although some mammals have different numbers of barrier layers, the placental diffusing capacity remains similar among these species [141]. During the first trimester of human pregnancy, maternal spiral arteries are clogged with trophoblast cells derived from the developing embryo, resulting in fetoplacental hypoxia. At this stage, hypoxia is not pathological, on the contrary, it drives the placental and initial foetal development [142]. Meanwhile, clogged maternal arteries undergo physiological remodelling. This is a crucial process to ensure adequate placental perfusion throughout pregnancy, since foetal oxygenation depends strongly on uteroplacental blood flow. During physiological vascular conversion, the endothelial lining and vascular smooth muscle layer are replaced by fibrinoid, leading to vascular lumen enlargement. Remodelled vessels cannot respond to vasoactive substances to the degree they did before the remodelling [143], meaning that maternal blood flows into the intervillous space more continuously and under lower pressure [144]. Such low-resistance flow protects the chorionic villi and provides adequate time for the exchange of nutrients and gases. Moreover, the pressure difference between the intervillous space and foetal capillaries affects the thickness of the villous membrane, thus influencing the placental diffusing capacity [144].

Abnormal placental development with poor spiral artery remodelling can adversely affect placental haemodynamics and placental diffusing capacity [139,145] and lower foetal oxygenation may result from abnormal villous development [146]. Together, the inadequate conversion of spiral arteries along with abnormal villous development can cause placental insufficiency and jeopardize foetal development [145]. The shallow, or even absent, trophoblast invasion of spiral arteries is considered one of the causes of preeclampsia [147]. In preeclampsia, insufficiently remodelled spiral arteries still have a muscle layer [148,149], so they are more reactive to vasoactive substances [80]. In addition, insufficient remodelling can be accompanied by placental atherosis, which is characterized by fibrinoid necrosis of the vessel wall [150] and thrombosis [151], both contributing to uteroplacental ischaemia-reperfusion injury and subsequent oxidative stress [139]. As a result, intermittent placental perfusion is more frequent throughout the pregnancy than is normal [139]. The intermittent perfusion becomes a problem, especially towards the end of a pregnancy, when fetoplacental oxygen consumption is at its peak [148].

Placental vessels lack innervation, and their reactivity mostly results from locally produced substances [80]. Typically, if shear stress is high, placental endothelial cells produce NO, so placental vascular resistance decreases; however, in placental vessels from growth-restricted foetuses, the shear stress-induced vasodilation is impaired, resulting in a parallel increase in placental vascular resistance [136]. The strongest vasoconstrictor produced by preeclamptic placental tissue is probably thromboxane, but other local substances are involved, such as Ang II and endothelin. Placental vasoreactivity is amplified by decreased prostacyclin and prostaglandin E2 production [80]. Finally, the chronic increase in adenosine concentrations in preeclampsia stimulates the production of anti-angiogenic soluble fms-like tyrosine kinase-1, a non-membrane associated splice variant of receptor 1 for vascular endothelial growth factor (VEGF). It binds the angiogenic VEGF, decreasing its free circulating concentrations and reducing vessel growth and placental vasculature [152].

An inappropriate oxygen environment can induce changes in the placental structure, which might be beneficial for improving foetal oxygenation. A placental barrier can become thinner, capillary diameter increases and uteroplacental vascular resistance decreases, so a more efficient diffusion is achieved [138]. These changes are mediated by HIF-1α target genes and their proteins, such as VEGF and erythropoietin [153]. Moreover, hypoxia can stimulate the expression of arginase-2, an enzyme responsible for decreased NO production [136]. Higgins et al. demonstrated in a murine model that there is an oxygen threshold below which the placenta cannot compensate for the lack of oxygen and intrauterine growth restriction occurs. Their experiment showed that an inhalation of 13% O_2_ during pregnancy led to structural changes in the placenta (reduction of the thickness of the interhaemal membrane, increased labyrinth zone volume, reduced trophoblast volume, increased placental capacity for transport of nutrients and O_2_ to the foetus), which spared foetal growth. On the other hand, when pregnant dams inhaled 10% O_2_, the placental barrier became thicker and the exchange surface area was reduced, so the placental diffusing capacity was negatively affected, and foetal growth restriction occurred.

Along with structural changes, placental metabolism is also modified by hypoxia. The hypoxic placenta consumes less oxygen but increases glucose transport and uptake for anaerobic glycolysis. The placenta is susceptible to oxidative stress due to its high metabolic activity; however, with increasing foetal oxygen requirements and increased metabolic activity, the antioxidant capacity of the placenta gradually increases [154]. Such reprogramming can protect the foetus from growth restriction [155], but if the transplacental glucose transport is decreased and placental consumption is still increased, the foetus becomes hypoglycaemic, resulting in growth restriction [153,156]. Two other factors can affect placental metabolism: the stage of pregnancy when hypoxia occurs and maternal food intake [155]. Hypoxia can modulate maternal food intake, therefore, it might be difficult sometimes to distinguish whether the effects are due to a lack of oxygen or a lack of nutrients. The same applies for glucocorticoids; hypoxia can also induce glucocorticoid secretion [157]. A foetus is protected from maternal glucocorticoids by the placental enzyme 11β-hydroxysteroid dehydrogenase (11β-HSD) type 2, converting the biologically active cortisol to the inactive cortisone. Hypoxia reduces the expression of 11β-HSD type 2 [81] and thus allows more glucocorticoids to cross the placental barrier. In contrast, hypoxia does not affect the expression of 11β-HSD type 1, which converts inactive cortisone to active cortisol [158]. Many articles have analysed the effect of antenatal glucocorticoid exposure on the development of the foetal cardiovascular system [159]. Antenatal glucocorticoids have direct and mediated (Ang II, catecholamines) pressor and morphological effects, such as cardiomyocytes maturation as well as growth and differentiation of the smooth muscle and endothelial cells in vessels [160]. Based on these findings, the placenta integrates multiple signals to compensate for the foetal demands. Whether these adaptations are beneficial for the foetus depends not only on the severity of hypoxia but also on other factors that may buffer or amplify the effects of hypoxia.

### 3.3. Mother

The foetus is entirely dependent on maternal oxygen supply and therefore, maternal hypoxaemia strongly affects the foetus. Maternal hypoxaemia can arise from different aetiologies, which are related to maternal health conditions, such as maternal haematological disorders, chronic pulmonary disease, and heart disease, or various environmental factors. Hypoxia is usually studied by inhaling low oxygen air, which mimics the environment at high altitudes. Millions of people live permanently in high-altitude environments and are expected to be genetically well adapted. Indeed, women living in high altitudes have a more significant increase in uterine perfusion during gestation and because of that, better foetal outcomes [161]. Maternal health conditions can result in an insufficient oxygen supply, even when the mother herself is not hypoxic. In such cases, foetal hypoxia could be a consequence of reduced uteroplacental perfusion or increased fetoplacental metabolism.

Gestational diabetes is one of the most common complications of pregnancy. According to the studied population, its prevalence ranges from 1.7% to 11.6% [162]. During pregnancy, the maternal metabolism changes to ensure optimal foetal development, and a pregnant woman becomes insulin resistant to provide the foetus with a sufficient amount of glucose. When the pancreas of a hyperglycaemic pregnant woman cannot produce enough insulin to maintain glycaemia [163], more glucose passes through the placental barrier to the foetus, which becomes hyperglycaemic. Consequently, foetal hyperinsulinaemia develops with a consequent increase in size and metabolism of the foetus [164]. These changes are reflected in increased uteroplacental and foetal oxygen consumption [165], and if fetoplacental demands exceed the maternal oxygen supply, hypoxia occurs [166]. Moreover, gestational diabetes is associated with a changing “zigzag” pattern of heart rate variability in the foetus [167]. Similar changes were observed during the second stage of parturition when an overstretched or compressed umbilical cord occurred, or during reduced oxygen availability because of uterine contractions [167]. The observed changes suggest that heart rate variability and the “zigzag” profile may be used as an early marker of prenatal hypoxia and not only in pregnancies with gestational diabetes [167].

Haematological disorders. Epidemiological data show that every fifth pregnant woman is anaemic and in developing countries, the prevalence might even reach 75% [168]. During the first trimester, a woman’s blood volume starts to expand, followed by a later increase in red blood cell mass. These pregnancy-induced changes result in physiological anaemia [169]. Since iron is needed for red blood cell formation, its deficiency reduces the capacity of blood to carry oxygen [170]. Interestingly, compensatory changes may lead to paradoxically higher oxygen content in the umbilical cord [171]. Another example of haematological disorders may be an abnormal, rigid sickle shape of erythrocytes, observed in thalassemia [64].

Pulmonary complications. Pregnancy-induced changes may also contribute to the development of obstructive sleep apnoea, whose prevalence reaches up to 26% in late pregnancy because of a higher maternal body mass index [172]. Obstructive sleep apnoea is characterized by episodes of hypopnoea or even apnoea resulting in maternal intermittent hypoxaemia and hypercapnia [173]. Even though such a shortage of oxygen is not necessarily transmitted to the foetus [174], data indicate the association between obstructive sleep apnoea and adverse foetal growth [175]. Among other respiratory disorders, asthma is the most common during pregnancy, with a worldwide prevalence of 2–13% [176]. When airways are obstructed, ventilation becomes uneven, and maternal arterial oxygen saturation decreases [177]. Acute respiratory diseases such as bronchitis, and pneumonia can lead to maternal respiratory failure, represent a risk for the foetus, and are a common pulmonary problem during pregnancy [64].

Cardiac diseases occur in 1% of pregnant women [178]. Maternal cardiac output increases throughout a normal pregnancy until reaches more than 30% of the non-pregnant value [179]. This physiological change, accompanied by a decrease in systemic resistance, is necessary to ensure adequate foetal oxygenation. If a pregnant woman suffers from heart disease, the heart may not adapt to this increased load [180] leading to arrhythmias, heart failure [181], and pulmonary oedema. In such cases, insufficient gas exchange in maternal lungs causes hypoxaemia also in the foetus [180].

Lifestyle. Maternal hypoxia can also develop due to bad lifestyle habits, such as a high-fat diet, smoking, or alcohol consumption. Consumption of a high-fat diet is associated with lower uteroplacental perfusion. Moreover, if a high-fat diet is accompanied by maternal hyperinsulinaemia, it can result in placental dysfunction [182] and the placental oxygen transport it might become limited. Moreover, obese women are more susceptible to developing the above-mentioned diseases, such as gestational diabetes or obstructive sleep apnoea [183], which may further increase the risk of hypoxia. Active smoking causes a rapid increase in maternal pulse and blood pressure. These cardiovascular changes result from the action of serum catecholamines, whose concentration increases within a few minutes after smoking [184]. When the uterine vessels are constricted, uteroplacental blood flow might become temporarily limited, while maternal concentrations of carboxyhaemoglobin also increase, resulting in a lower oxygen supply for the foetus, thus making hypoxia even more pronounced [184]. A recent study showed that passive smoking might also cause foetal hypoxia [185]. Alcohol consumption results in placental oxidative stress and a subsequent decrease in NO availability [186]. Even drinking coffee during pregnancy could potentially affect foetal oxygenation by stimulating the maternal and placental renin-angiotensin system [187] and maternal catecholamine secretion [188].

## 4. Factors Affecting the Consequences of Hypoxia

In general, the effects of prenatal hypoxia depend on several variables, such as the duration of prenatal hypoxia, environmental factors, sex of the foetus, stage of development, and phase of the day when hypoxia occurs.

### 4.1. Sex

Animal studies suggest a sex-dependent susceptibility to cardiovascular disease in adulthood after exposure to prenatal insults. Female offspring possess protective mechanisms against prenatal hypoxia programming effects, while male offspring have an increased susceptibility to cardiovascular diseases [189] and the negative consequences of prenatal hypoxia are manifested differently at the local and systemic levels depending on sex.

Another sex-dependent effect of prenatal hypoxia is the vasoactive response of vessels to endothelin-1. The mesenteric arteries of male rats exposed to prenatal hypoxia show an increased vasoconstrictive response to endothelin-1 as compared with that of female rats. The administration of an endothelin-1 antagonist results in a significantly greater blood pressure reduction in males as compared with that in females [39]. Sex-dependent changes were also observed in the ryanodine receptor 2 in the heart because prenatal hypoxia increased the ryanodine receptor 2 in males, but not in females [190]. Moreover, males have a higher susceptibility to the development of hypertension at an earlier age [9,94] and have an increased susceptibility to ischaemia/reperfusion injury in comparison with females [16]. This difference can be explained by decreased protein kinase Cε activity through an epigenetic modification [10], because this enzyme is important in cardioprotection against ischaemia/reperfusion injury [191].

The kidney of the male foetus is more sensitive to hypoxia than that of the female foetus, because no glomerular loss or renal fibrosis was observed in the hypoxic female rat foetus. Males have more glomeruli than females under basal physiological conditions, and prenatal hypoxia causes a reduction up to 25% compared with controls [94]. Renin expression is higher in juvenile male offspring exposed to prenatal hypoxia than in females but without differences in the AT_1_ receptor and angiotensin-converting enzyme gene expression in the kidney [94]. The difference in the renal response to prenatal hypoxia between females and males suggests that mechanisms exist in females that protect the kidney from prenatal hypoxia at an early age.

Chronic prenatal hypoxia (14% O_2_; ED 6–18) in offspring male mice (8-months-old) led to an increased ROS production and lower respiratory capacity, which was associated with a reduced protein expression of mitochondrial complex I, II, and IV. In females of the same age, the opposite effect was observed, with a higher respiratory capacity and lower ROS production that was associated with the increased enzymatic activity of complex IV [44]. Prenatal hypoxia can be experimentally treated by MitoQ, which is a mitochondria-targeted antioxidant used for the treatment of oxidative stress. MitoQ has protective effects in the foetus of males and females, increasing placental efficiency and decreasing placental superoxide concentration, but only an improved oxygenation and normalized hypoxia-induced gene expression in females [192]. Thus, it seems that MitoQ has more protective effects in females than in males.

Ovariectomy and castration reduce the sex differences in blood pressure due to placental insufficiency [193]. Placental insufficiency in rats led to intrauterine growth restriction and increased blood pressure in 3-months-old males, while females of the same age were normotensive [194]. In the case of plasma oestrogen levels, no differences were observed between females prenatally exposed to hypoxia and controls [195]. Ovariectomy in females exposed to prenatal hypoxia increased the blood pressure response to Ang II, which is normally reduced due to prenatal hypoxia; however, oestrogen administration did not affect the blood pressure response in hypoxic females. The role of the ovary in regulating blood pressure and cardiovascular function is probably related to oestrogen and its downstream signalling pathways and its relationship with other hormones [195]. Despite apparent ambiguities, the oestrogen receptors are involved in cardio-protection. Oestrogen receptor stimulation leads to eNOS activation, AT_1_ receptor up-regulation and AT_2_ receptor down-regulation, while oestrogen receptor knockouts reduce cardio-protection and increase the susceptibility to injury from ischemia/reperfusion injury [196]. These effects are age-dependent, because in older ovariectomised rats, oestrogen supplementation decreases the AT_1_ receptor and increases the AT_2_ receptor expression [196].

The role of androgen in prenatal programming is less understood. In 12-months-old females with intrauterine growth restriction, increased values of plasma testosterone and blood pressure were observed, and blockade of the androgen receptor decreased blood pressure [197]. Similar to oestrogen, testosterone interacts with the RAAS, while androgen receptor blockers decrease the AT_1_ receptor and angiotensin-converting enzyme [198]. Moreover, enalapril (an angiotensin-converting enzyme inhibitor) suppressed the increase of blood pressure in a intrauterine growth restriction female, however this was less significant if the androgen receptor was blocked. A similar effect was observed in males [197]. Serum testosterone and oestradiol were increased in male rats due to prenatal hypoxia [199] and castration in these animals reduced blood pressure, while an administration of enalapril caused a more pronounced decrease in blood pressure in intact males compared with castrated rats [198]. This knowledge points to the complexity of prenatal programming and its effect in sex-dependent manners.

### 4.2. Circadian Variability

The effects of prenatal hypoxia on the cardiovascular system can also be modified by the circadian system because all important organs (kidneys, heart, and blood vessels) are under the circadian control of the master circadian oscillator localised in the suprachiasmatic nuclei in the hypothalamus of the brain. Moreover, all these organs, as well as nearly all cells in the body, contain peripheral oscillators, which are synchronised through humoral and neural pathways [200]. The central and peripheral oscillators generate circadian oscillations based on the rhythmic expression of so-called clock genes (*Clock*, *Bmal1*, *Cry1*, *Per1*, *Per2*, *Per3*, *Dec1*, *Dec2*, and *Rev-erba*). This molecular mechanism is based on the complex transcription–translation feedback loops, in which the transcriptional factors BMAL1 and CLOCK govern the rhythmic expression of negative factors encoded by genes *Per1,2,3* and *Cry1* and *2*, which feedback and inhibit the expression of the *Bmal1* and *Clock* genes [201,202]. Heterodimer BMAL1 and CLOCK also activates the expression of *Rev-erba,* which protein inhibits *Bmal1*. Similarly, *Dec1* and *Dec2* are transactivated by heterodimer [203]. Circadian rhythms are endogenously generated and synchronised predominantly by the light–dark cycle to rhythmic environmental conditions, but other cyclic changes, such as feed intake, can efficiently synchronise the peripheral organs, such as the liver [204,205]. During the gravidity, clock gene expression is significantly changed [206].

The endogenous circadian rhythms in the foetus develop gradually and start to function autonomously only after birth [207]. Nutrients and maternal humoral factors, such as melatonin and glucocorticoids are important synchronizing factors for the foetus [200,208]. Maternal corticosterone levels increase significantly during gravidity and exhibit circadian rhythms, which are lost at the end of gravidity [206]. Glucocorticoids can set a circadian clock in the foetus and also accelerate the development of the circadian oscillator and circadian rhythmicity [208]. Moreover, the placental enzyme 11β-HSD type 2, which has the important mediatory function regarding the actions of glucocorticoids, is directly regulated by clock genes. The activity of 11β-HSD type 2 is highest in the morning, corresponding to the peak of maternal glucocorticoid secretion [209]. Thus, such an activity pattern protects the foetus from excessive glucocorticoid exposure [209]. In addition, the expression of placental glucose and amino acid transporters may also show a circadian pattern [210], suggesting that desynchronization might probably affect placental nutrient transport. Moreover, other regulatory mechanisms maintaining the homeostasis of the cardiovascular system, such as NO, autonomic nervous system and RAAS, exhibit distinct circadian rhythms. In the foetus, heart rate has a significant daily rhythm, which is modified by sex, maternal locomotor activity and season [211]. The rhythm is clearly imposed by maternal factors, but their interplay with the development of the circadian system of the foetus and other factors, which can interfere with them, can have clinical significance [211].

Gestational hypoxia can regulate trophoblasts’ proliferation, migration, and invasion ability through *Clock* expression. The expression of *Clock* in the placenta was significantly higher in a prenatal hypoxia group than in controls, and the silencing of *Clock* caused an improvement of the proliferation, migration, and invasion ability of trophoblasts. Since *Clock* is more stable than *Hif*, it was suggested as a more reliable marker of the hypoxia in the placenta [212]. Moreover, recent studies indicate that manipulating the clock genes can act as a therapeutic tool to minimize the pathologies associated with hypoxia [62].

#### 4.2.1. Hypoxia-Inducible Factors

**Figure 2:**
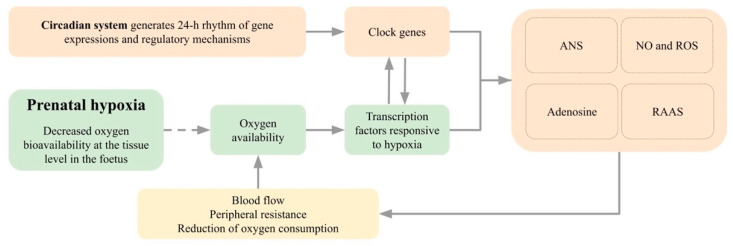
Interaction between prenatal hypoxia and the circadian system. Prenatal hypoxia decreases oxygen bioavailability in the tissues of the foetus. The sensitivity of the cell to decreased oxygen supply is mediated by hypoxia-inducible transcription factors, which interact with multiple physiological systems, including bi-directional interaction with the circadian system. Transcription factors induced by hypoxia show daily rhythms and directly bind to the E-box of the clock genes, thereby modulating the function of the circadian system. Transcriptional factors that respond to hypoxia also interact with the cardiovascular regulatory mechanisms to maintain homeostasis and sufficient blood flow through vital organs by increased peripheral resistance and reduction of oxygen consumption. ANS, autonomic nervous system; NO, nitric oxide; ROS, reactive oxygen species; RAAS, renin-angiotensin-aldosterone system.

Circadian clocks cause daily oscillations of oxygen levels under physiological conditions with increased consumption during the active phase of the day, which is associated with physical activity and food intake, because both activities require high oxygen consumption [213]. Likewise, organ oxygenation is rhythmic, reaching peak levels during the active phase [214]. Moreover, oxygen is an important synchronizing factor for the circadian rhythm. Many studies suggest a two-way relationship between the transcription factors responding to hypoxia and clock genes [215,216] (Figure 2). The activity of HIF-1 is regulated by circadian clock components and the increased expression of PER2 stabilises and increases HIF-1 activity. In addition, hypoxia increased the expression of Dec1, which is also enhanced by light [203]. The reciprocal relationship between HIFs and the circadian clock is considered a therapeutic target and can contribute to the development of new therapeutic approaches [217].

Stable HIF-1 binds to the hypoxia response element, which is found in the promoter or enhancer region of hundreds of genes, thereby increasing the transcription of these target genes above a basal level. Moreover, HIF-1α may be able to bind to the E-box of clock genes and influence the transcription–translation feedback loop [218]. Studies suggest that the physiological rhythms of oxygen reset the molecular clock in cultured cells [214]. In *Hif-1α* deficient cells, the oxygen rhythms did not induce cyclic expression of the clock genes, and the expression levels of clock genes were consistently low compared with control cells, except for *Clock*, which was increased in the *Hif-1α* deficient cells. This suggests that HIF-1α represents a link between oxygen and the circadian clock [219]. Exposure to intermittent hypoxia in adult mice affects clock genes’ expression more in the central (the brain) than in the peripheral tissue (the liver). In the brain, *Arntl* and *Nr1d1* expression became arrhythmic compared to *Cry2* expression. The expression of *Per1* and *Per2* resulted in their lower amplitude after hypoxia. In the liver, *Per1* and *Nr1d1* rhythms had a reduced amplitude, while *Clock* became arrhythmic [220]. The effects of hypoxia on the circadian clock were not analysed in the prenatal period; however, prenatal maternal stress had long-lasting effects on the circadian system in offspring, because desynchrony among individual SCN neurones was observed and canonical clock gene rhythms were impaired in the central oscillator and peripheral tissues [221]. The results suggest that the effects of hypoxia on canonical clock genes are tissue-specific and illustrate the way in which hypoxia can exert its influence on health in adulthood.

On the other hand, the effects of prenatal hypoxia can be modified by circadian regulation because the clock genes regulate the transcription factors induced by hypoxia (Figure 2). The transcript of *Hif-1α* is constant, while the nuclear protein HIF-1α levels in the kidneys and brain have circadian rhythms [214]. An analysis of organs, such as the liver, kidneys, and lungs, revealed that the lowest time-dependent transcription response was in the lungs. A similar response to hypoxia was observed in conditions without synchronizing factors; therefore, it is assumed that the transcription response to hypoxia is endogenously regulated [213]. This response was affected in mice with disrupted circadian systems, and the time-dependent response was lost [213].

Plasticity and critical windows in prenatal development are regulated across multiple scales, from milliseconds and minutes, controlled by neuronal oscillations, up to oscillations with a key role of the clock genes. Moreover, plasticity windows are sensitive to circadian gene manipulation [222]. These findings support the theory that the effects of prenatal hypoxia on the foetus are time- and circadian-dependent. 

Hypoxia plays an important role in developing many cardiovascular diseases, which often exhibit the daily rhythms, and the interaction between HIFs and clock genes can have a significant role in this process [218]. Relationship between the HIFs and circadian rhythmicity was also observed in mice with myocardial infarction. Consequences of myocardial infarction were circadian-dependent and varied throughout the day. Mice with *Per1*^−/−^ and *Per2*^−/−^ deletion showed more severe effects than the controls with functional clock machinery. Thus, the clock genes play an important role in regulating and expressing HIF-target genes during hypoxic conditions [62]. In this line, circadian dependent programming effects were observed under hyperoxic conditions during early life. Mice infected by the influenza virus at the end of the passive phase had significantly higher mortality than mice infected at the end of the active phase of the day [223], while mice exposed to hyperoxia during early postnatal life exhibited reduced circadian-dependent mortality due to influenza [224]. This effect can be explained by a loss of circadian rhythmicity in the lungs. It can be assumed that prenatal hypoxia acting during different times of the day may affect the regulatory mechanisms of the cardiovascular system to different extents because the transcription factors responding to hypoxia have different levels of expression and activity over the 24 h period; however, all studies analysed the effect of chronic prenatal hypoxia during the light (passive) phase in nocturnal rodents, and more studies exploring these effects during the night are needed.

#### 4.2.2. Consequences

Prenatal hypoxia can modulate the circadian system and thus may affect the reactivity to challenges and susceptibility to diseases later in adulthood. Chronic prenatal hypoxia in rats can induce changes in the circadian rhythm of locomotor activity. For example, locomotor activity in rats prenatally exposed to hypoxia was phase-advanced, and the animals were less active than controls [24]. When animals were exposed to the new light regimen, the time required for resynchronization was prolonged in prenatally hypoxia-affected rats, while no difference was observed during constant darkness. Thus, prenatal hypoxia had no effects on the endogenous rhythms. This may indicate that the chronic prenatal hypoxia affects the synchronization to environmental stimuli and the physiological and behavioural responses to light in adulthood [24]. Exposure of rats to intermittent 4 h periods of hypoxia on ED 19 and ED 20 during the daytime (passive phase for rats) increased the blood pressure in adult male offspring but did not affect the circadian rhythms of blood pressure and heart rate [14]. Similarly, prenatal hypoxia (12 h, ED 20) during the light phase did not change the circadian rhythms of blood pressure and heart rate in male offspring, as well as the response of the cardiovascular system to vasoconstriction drugs [30,132]; however, these animals had an altered response to artificial light at night [132], which is considered as a risk factor for the disruption of circadian control and the development of cardiovascular diseases [225].

Prenatal hypoxia can program and negatively determine the development of hypertension in adulthood differently relating to the stages of prenatal development. Therefore, knowledge of the critical developmental stages is essential because it allows for changing the homeostatic set points and dynamic ranges of physiological systems, thus preventing the development of hypertension in adulthood [226]. Many biochemical and physiological processes develop circadian oscillations during the perinatal period. Even short-term hypoxia during the passive phase of the day in rats can have significant effects on the cardiovascular system in adulthood; however, there are no studies analysing the effects of prenatal hypoxia during the active phase of the day and more studies in this field are needed. We assume that the circadian aspects should be important for understanding the effects of prenatal hypoxia on the cardiovascular system.

## 5. Conclusions

Prenatal hypoxia affects the foetal cardiovascular system, resulting in the development of hypertension and cardiovascular diseases later in adulthood (i.e., prenatal programming). The consequences of prenatal hypoxia are often associated with intrauterine growth restriction; however, even short-term prenatal hypoxia can program hypertension of offspring without affecting birth body weight. As a result of prenatal hypoxia, the foetus activates complex adaptive regulatory mechanisms, including the chemoreflex, autonomic nervous system, NO, ROS, adenosine, catecholamines, and RAAS, which can be modulated by placental and maternal humoral factors. The placenta has a key role, and it can compensate for a low partial pressure of oxygen, thus maintaining the demands of the foetus. Therefore, abnormal placental development contributes to prenatal hypoxia. Oxygen supply to the foetus is entirely dependent on the mother, and prenatal hypoxia can also occur due to a mother’s health condition or lifestyle. The duration of hypoxia, stage of development, sex of progeny, and phase of the day contribute to the prenatal programming of the cardiovascular system in adulthood. Prenatal hypoxia has more severe effects on male offspring compared with female offspring. Until now, there is limited information on prenatal hypoxia occurring during different times of the day on the homeostatic plasticity of the cardiovascular system development. If the sensitivity of the developing systems depends on the time of day, this opens new ways for understanding the mechanisms of prenatal programming and opportunities to prevent the development of cardiovascular hypertension in adulthood.

## Data Availability

Not applicable.
